# GC-MS Chemical Profiling, Biological Investigation of Three *Salvia* Species Growing in Uzbekistan

**DOI:** 10.3390/molecules27175365

**Published:** 2022-08-23

**Authors:** Haidy A. Gad, Rano Z. Mamadalieva, Noha Khalil, Gokhan Zengin, Basma Najar, Olim K. Khojimatov, Nawal M. Al Musayeib, Mohamed L. Ashour, Nilufar Z. Mamadalieva

**Affiliations:** 1 Department of Pharmacognosy, Faculty of Pharmacy, Ain Shams University, Cairo 11566, Egypt; 2 Kokand State Pedagogical Institute, Kokand 713000, Uzbekistan; 3 Department of Pharmacognosy and Medicinal Plants, Faculty of Pharmacy, Future University in Egypt, Cairo 11835, Egypt; 4 Department of Biology, Science Faculty, Selcuk University, Konya 42130, Turkey; 5 Department of Agriculture, Food and Environment, University of Pisa, Via del Borghetto 80, 54126 Pisa, Italy; 6 Institute of Botany, Academy of Sciences of the Republic of Uzbekistan, Tashkent 100125, Uzbekistan; 7 Department of Pharmacognosy, College of Pharmacy, King Saud University, Riyadh 11451, Saudi Arabia; 8 Institute of the Chemistry of Plant Substances, Academy of Sciences of the Republic of Uzbekistan, Tashkent 100170, Uzbekistan

**Keywords:** antioxidants, chemical profile, chemometric analysis, enzyme inhibition, GC-MS, *Salvia*

## Abstract

*Salvia* is a potentially valuable aromatic herb that has been used since ancient times. The present work studied the chemical profile of three *Salvia* species essential oils (EO): *S. officinalis*, *S. virgata* and *S. sclarea*, as well as assessing their antioxidant and enzyme inhibitory activities. A total of 144 compounds were detected by GC-MS analysis, representing 91.1, 84.7 and 78.1% in *S. officinalis*, *S. virgata* and *S. sclarea* EOs, respectively. The major constituents were *cis*-thujone, 2,4-hexadienal and 9-octadecenoic acid, respectively. The principal component analysis (PCA) score plot revealed significant discrimination between the three species. The antioxidant activity of the EOs was evaluated using in vitro assays. Only *S. virgata* EO showed antioxidant activity in the 2,2-diphenyl-1-picryl-hydrazyl (DPPH) assay (26.6 ± 1.60 mg Trolox equivalent (TE)/g oil). Moreover, this oil exhibited the highest antioxidant activity in 2,2-azino bis (3-ethylbenzothiazoline-6-sulphonic acid) (ABTS), cupric-reducing antioxidant capacity (CUPRAC) and ferric-reducing power (FRAP) assays in comparison with the other two EOs (190.1 ± 2.04 vs. 275.2 ± 8.50 and 155.9 ± 1.33 mg TE/g oil, respectively). However, *S. virgata* oil did not show any effect in the chelating ability assay, while in the PBD assay, *S. officinalis* had the best antioxidant activity (26.4 ± 0.16 mmol TE/g oil). Enzyme inhibitory effect of the EOs was assessed against acetylcholinesterase (AChE) and butyrylcholinesterase (BChE), tyrosinase, α-glucosidase and α-amylase. AChE enzyme was more sensitive to *S. officinalis* EO (4.2 ± 0.01 mg galantamine equivalent (GALAE)/g oil), rather than *S. virgata* EO, which was ineffective. However, *S. virgata* had the highest BChE effect (12.1 ± 0.16 mg GALAE/g oil). All studied oils showed good tyrosinase inhibitory activity, ranging between 66.1 ± 0.61 and 128.4 ± 4.35 mg kojic acid equivalent (KAE)/g oil). Moreover, the EOs did not exhibit any glucosidase inhibition and were weak or inefficient on amylase enzyme. Partial least squares regression (PLS-R) models showed that there is an excellent correlation between the antioxidant activity and the volatile profile when being compared to that of enzyme inhibitory activity. Thus, the studied *Salvia* essential oils are interesting candidates that could be used in drug discovery for the management of Alzheimer’s and hyperpigmentation conditions.

## 1. Introduction

*Salvia*, a popular aromatic plant known as sage, is an evergreen perennial subshrub native to the Mediterranean region and cultivated in several parts of the world [1]. Genus *Salvia* L. is dominant in family Lamiaceae and comprises around 900 species [2]. The word *Salvia* in Latin means “healthy” or “to heal”, which indicates the plethora of notable uses due to the variety of biologically active metabolites present in this plant.

Sage was popular in Egyptian, Greek and Roman medicine [3]. Ancient Egyptians used the leaf to enhance fertility, while the Greeks used it to treat cough, enhance memory, and heal ulcers, sores and wounds. The plant is widespread in many cultures due to its culinary, medical and psychological effects. It is usually used as herbal tea, oil, flavor in cosmetics, perfumery and pharmaceutical products. Traditionally, it has been used in treating malaria, microbial infections, as a home disinfectant, mood elevator, to enhance cognitive performance and in managing some gastrointestinal disorders such as dyspepsia, spasms and flatulence [4,5].

Numerous studies have reported the essential oil yield and composition of different *Salvia* species. The variation of yield and composition is attributed to several factors, mainly environmental and agronomic conditions [6]. The essential oil has shown cytotoxic [7], antimutagenic [8], antimicrobial [9], hepatoprotective [10] and neuroprotective effects in addition to the treatment of some neurodegenerative disorders such as Alzheimer’s disease [11].

*S. officinalis* is the most popular species within the *Salvia* genus. It has traditionally been used to improve cognitive function and skin care [12]. Similarly, *S. virgata* has also been used in treating some skin diseases and for wound healing [13]. On the other hand, *S. sclarea* has been used as herbal tea as a tranquillizer and to improve circulation. Its oil has been used as an antimicrobial and food preservative [14].

The present study aimed to investigate the chemical profile of the essential oils of three *Salvia* species growing in Uzbekistan, *S. officinalis* L. (local Uzbek name is Dorivor marmarak)*, S. virgata* Jacq. (Zig’irak marmarak) and *S. sclarea* L. (Mavrak). Principal component analysis (PCA) and hierarchical cluster analysis (HCA) were employed to differentiate between the three species based on the chemical profile of their essential oils. Additionally, the antioxidant activity of the oils was assessed using in vitro assays, as well as the enzyme-inhibitory activities against five enzymes that are crucial in certain diseases such as diabetes, Alzheimer’s and hyperpigmentation.

## 2. Results and Discussion

### 2.1. GC-MS Analysis of the Essential Oils of Salvia Species

GC-MS analysis of the oils could detect 144 compounds in the three oils, representing 91.1, 84.7and 78.1% of *S. officinalis, S. virgata* and *S. sclarea* EOs, respectively (Table 1) supplementary materials Figure S1 in the Supplementary Material. In *S. officinalis* oil, the major compounds were *cis*-thujone (18.6%), camphor (12.2%), 1,8-cineole (8.9%), α-humulene (6.1%) and *n*-butyl octadecenoate (5.6%). *S. virgata* EO was characterized by 2,4-hexadienal (9.4%), limonene (6.2%), γ-terpinene (5.2%) and *p*-cymene (4.5%), while in *S. sclarea*, 9-octadecenoic acid was the main constituent (6.9%), followed by *n*-butyl octadecenoate (5.7%) and linalyl acetate (4.7%). Two phenylpropanoids, eugenol and methyl eugenol, were detected with varying percentages in *S. officinalis* and *S. sclarea* oils.

Several factors may affect essential oil composition, such as geographical origin, harvesting season, method of oil extraction and growing conditions [15]. Iranian *S. officinalis* evidenced α-thujone (37.2%) as the main constituent, followed by 1,8-cineole (12.7%) and β-thujone (9.1%) [16]. Tunisian *S. officinalis* EO was characterized by camphor (33.6%), 1,8-cineole (22.2%) and α-thujone (21.4%) [17]. Romanian *Salvia* collected from cultivated and commercial samples showed α-thujone as the major compound in all analyzed oil samples (31.2–52.8%), followed by camphor and viridiflorol [18]. All the cited compounds were present in the herein studied *S. officinalis* EO. α-Thujone was usually common as one of the major identified compounds in *S. officinalis* oil. This compound showed potent antioxidant activity in several in silico and in vitro assays, comparable to standard antioxidant agents such as ascorbic acid and Trolox [19]. It also significantly adjusted cholesterol and triglyceride levels in diabetic rat models [20].

Pentacosane (20.1%), caryophyllene oxide (6.9%), phytol (6.8%), spathulenol (6.1%) and nonacosane (5.2%) were chief compounds in Turkish *S. virgata* EO [21]. The Iranian *S. virgata*’s EO is typified by β-caryophyllene, caryophyllene oxide and spathulenol [22,23]. The reported studies were significantly different from our results.

This divergence was also noted in the chemical composition of the essential oil of *S. sclarea*, where it is reported that in the Polish species, linalool (42.3%), α-terpineol (13.4%), geraniol (6.3%) and geranyl acetate (5.4%) were prevailing compounds [24]. However, linalyl acetate (19.7−31.0%), linalool (18.5−30.4%), geranyl acetate (4.4−12.1%) and α-terpineol (5.1−7.6%) were major components in different samples collected from Greece. Leaf EO was characterized by sclareoloxide (27.3%), thymol (20.6%) and caryophyllene oxide (9.9%), while sclareol (33.9%), linalool acetate (10.6%) and manoyl oxide (9.6%) were identified as the main components in flower essential oil from Egyptian plants [25]. However, in the present study, sclareol was not detected, only its derivative sclareoloxide (1.5%).

### 2.2. Antioxidant Effect of the Essential Oils of Salvia Species

Six in vitro assays were employed to evaluate the antioxidant activity of the three *Salvia* EOs. These were radical scavenging activity using 2,2-diphenyl-1-picryl-hydrazyl (DPPH), 2,2′-azino-bis(3-ethylbenzothiazoline-6-sulfonic acid) (ABTS) radical cation-based assay, total antioxidant capacity using cupric-reducing antioxidant capacity assay (CUPRAC), ferric-reducing antioxidant power assay (FRAP), EDTA chelating activity and phosphomolybdenum (PBD) assay. Only *S. virgata* oil showed antioxidant activity in DPPH assay (26.6 ± 1.60 mg TE/g oil, IC_50_: 1.98 ± 0.23 mg/mL). Moreover, the same oil exhibited the highest antioxidant activity in ABTS, CUPRAC and FRAP assays with the lowest IC_50_ values (0.75 ± 0.02, 0.39 ± 0.02 and 0.28 ± 0.01 mg/mL, respectively) in comparison with the others. In addition, the essential oil was more active than Trolox (IC_50_: 0.44 ± 0.02 mg/mL) in CUPRAC. However, *S. virgata* oil did not show any effect in the metal chelating assay, while in PBD assay, *S. officinalis* had the best antioxidant activity (26.4 ± 0.16 mmol TE/g oil, IC_50_: 0.10 ± 0.01 mg/mL) (Table 2). All tested essential oils exhibited stronger abilities in PBD assay compared to Trolox (IC_50_: 0.68 ± 0.01 mg/mL).

It has been noted that natural products with antioxidant potential represent promising therapies for various diseases since excessive production of free radicals and lipid peroxidation of cell membranes are involved in the mechanistic pathophysiology of certain ailments, especially cardiovascular diseases, diabetes, Alzheimer’s, various types of cancers and others [26]. It is always recommended to assess the antioxidant activity of natural products by different methods with different mechanisms due to the complex nature of natural compounds [27]. Antioxidant activity of *Salvia* essential oils may be attributed to their volatile components. In the present study, it was found that *S. officinalis* oil is rich in oxygenated monoterpenes, which have been proven to possess the strongest antioxidant capacity relative to other classes of volatile compounds [28]. The major identified compound in this oil, α-thujone, showed good to moderate antioxidant capacity in a concentration-dependent manner in various assays such as DPPH, FRAP and hydroxyl, superoxide and nitric oxide radical scavenging activity [29]. A study showed that the antioxidant capacity of *S. officinalis* oil (with major compounds camphor and 1,8-cineole) was influenced by the time of hydro-distillation. The highest DPPH radical scavenging activity was observed for oil distilled in 2 h, while the highest activity in the TBARS assay was for oil distilled in 30 min. [30]. Regarding *S. virgata,* its flower oil showed better DPPH radical scavenging activity than its leaf oil, with activity equal to the standard butylated hydroxyanisole (BHA) [31]. Moreover, it was observed that oil isolated from aerial parts of *S. virgata* had better antioxidant activity in DPPH and FRAP assays when using the oil of full flowering rather than pre-flowering stage [32]. To the best of our knowledge, no previous extensive evaluation of the antioxidant activity of *S. sclarea* oil has been performed. However, its antioxidant capacity may also be attributed to some of its volatile constituents such as linalyl acetate, which has previously proven antioxidant potential in different assays, either in pure form or in oils where it is found as a major compound [33]. In addition to the different levels of different chemical components in the tested essential oils, the interactions between these components, namely antagonistic and synergetic, could affect the observed antioxidant properties [34,35,36].

### 2.3. Enzyme Inhibitory Effects of the Essential Oils of Salvia Species

The enzyme inhibitory effect of the oils was assessed against five enzymes which play a crucial step in certain medical conditions. Highest AChE inhibitory activity was recorded for *S. officinalis* (4.3 ± 0.01 mg galantamine equivalent (GALAE)/g oil; IC_50_: 0.68 ± 0.01 mg/mL), while *S. virgata* showed no effect at all. However, *S. virgata* had the highest BChE effect (12.1 ± 0.16 mg GALAE/g oil; IC_50_: 0.60 ± 0.01 mg/mL). All studied oils showed good tyrosinase inhibitory activity ranging between 66.1 ± 0.61 using *S. sclarea* EO to 128.4 ± 4.35 mg kojic acid equivalent ((KAE)/g oil) with *S. officinalis* EO. In addition, *S. officinalis* (IC_50_: 0.73 ± 0.01 mg/mL) exhibited stronger tyrosinase ability than standard inhibitor, kojic acid (IC_50_: 0.75 ± 0.01 mg/mL). Moreover, the oils did not exhibit any glucosidase inhibition, and exhibited weak or no activity as amylase inhibitors (Table 3).

Inhibition of AChE leads to the accumulation of acetylcholine, leading to better communication between nerve cells, and thus eases the symptoms in Alzheimer’s patients [37]. BChE is also a co-regulator of acetylcholine. Therefore, its inhibition leads to better symptoms and prognosis in Alzheimer’s [38]. Previous clinical studies showed that administration of sage oil and herbal teas improved mental and cognitive function in Alzheimer’s individuals [39]. Alcoholic extracts of *S. officinalis* exhibited in vitro inhibition of AChE and BChE, with higher inhibition observed against BChE [40], which is in accordance with the present results, but regarding the essential oil.

Tyrosinase is a rate-limiting enzyme in melanin biosynthesis, as it oxidizes the amino acid tyrosine into melanin [41]. Its inhibitors, such as kojic acid, ellagic acid and hydroquinone, are used in the treatment of hyperpigmentation conditions and in skin-whitening cosmetics. A study on 19 essential oils showed that *S. officinalis* oil had moderate tyrosinase inhibitory activity with IC_50_ 99.8 ± 1.750 μg/mL relative to kojic acid with IC_50_ 2.3 ± 0.054 μg/mL [42]. Regarding *S. virgata* and *S. sclarea* oils, no previous data on their tyrosinase inhibitory activity were reported.

Both α-glucosidase and α-amylase digest carbohydrates, which leads to increasing levels of postprandial blood glucose, and their inhibition would lead to controlling postprandial hyperglycemia in diabetic patients, as well as reducing the risk for developing diabetes [43]. Although the studied *Salvia* oils showed no α-glucosidase inhibition and weak or no activity as α-amylase inhibitors, however, previous reports regarding their alcoholic and aqueous extracts recorded inhibitory activity for those enzymes [44]. Thus, their antidiabetic activity may be attributed to other active constituents not present in their essential oils, such as phenolic compounds.

Taken together, the observed enzyme inhibitory effects of the *Salvia* essential oils could have great potential for further pharmaceutical, nutraceutical and cosmeceutical applications. However, due to the complex nature of essential oils, interactions between chemical components should not be forgotten [45,46,47].

### 2.4. Chemometric Analysis

**Figure 1:**
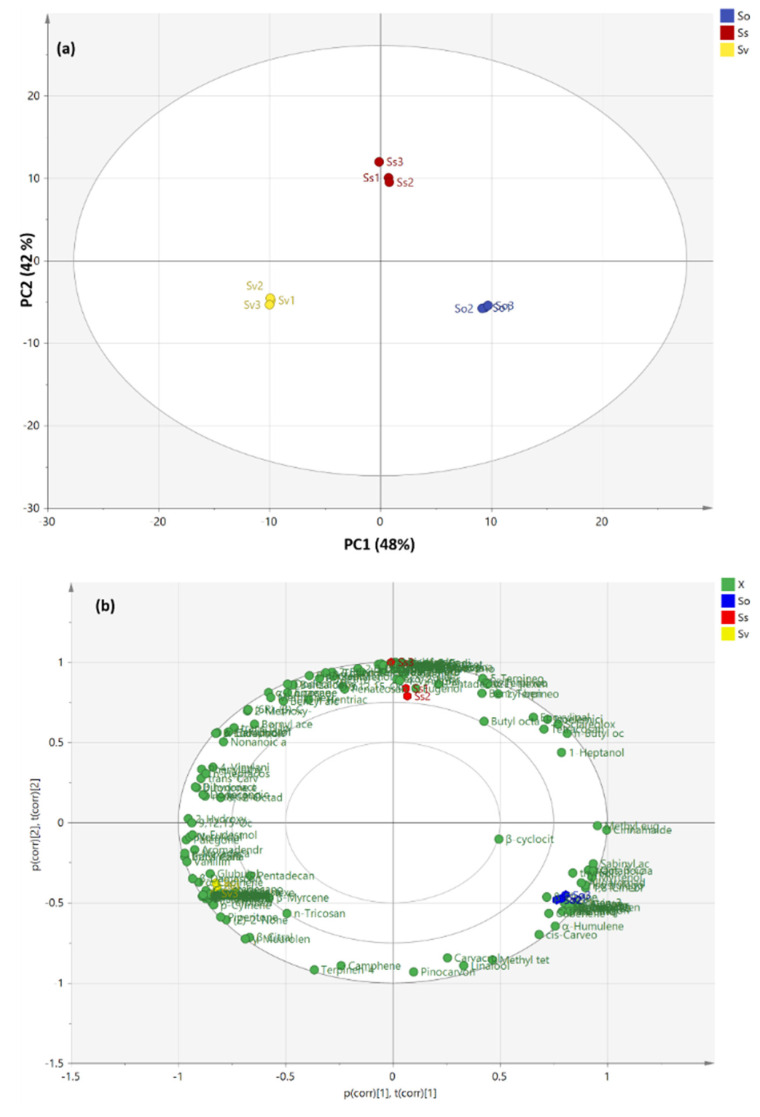
PCA score plot: (**a**) biplot; (**b**) based on GC-MS chemical profile of the essential oils of different *Salvia* species as displayed in Table 1. *S. officinalis* (So), *S. virgata* (Sv) and *S. sclarea* (Ss).

The GC-MS-based chemical profile of essential oils included both qualitative and quantitative discrepancies among different *Salvia* species; chemometric analysis was applied using principal component analysis (PCA) and hierarchal cluster analysis (HCA) to segregate closely related species, as well as to recognize any significant association between them [48]. A matrix of the total number of samples and their replicates (9 samples) multiplied by 144 variables (GC-MS peak area %) was constructed in MS Excel^®^, then subjected to chemometric analysis (PCA and HCA). Due to the large number of variables, PCA was first used to reduce the dimensionality of the multiple dataset, followed by removing the redundancy in the variables and utilizing raw data (peak area % for each compound as in Table 1). The PCA score plot accounting for 90% of the variation in the dataset (Figure 1a) highlights that the first principal component (48%) discriminates between *S. virgata* (Sv) (PC1 negative values on the lower quadrant) and the other two species (PC1 positive values), while the second principal component (42%) discriminates between *S. sclarea* (Ss) (positive loading along PC2) and the others (negative loading along the same axis).

Figure 1b displays the biplot for both scores and loading; the plot enabled the visualization of similarities and difference among different species in terms of their chemical profiles. The species sited near different metabolites are patterned in the score plot on the bases of these metabolites. The biplot shows that there is no specific marker (compound) accounting for the discrimination between *Salvia* species, proving the significant importance of the whole chemical profile of the essential oils in the discrimination between different species, not solely the compounds existing in high percentage.

**Figure 2:**
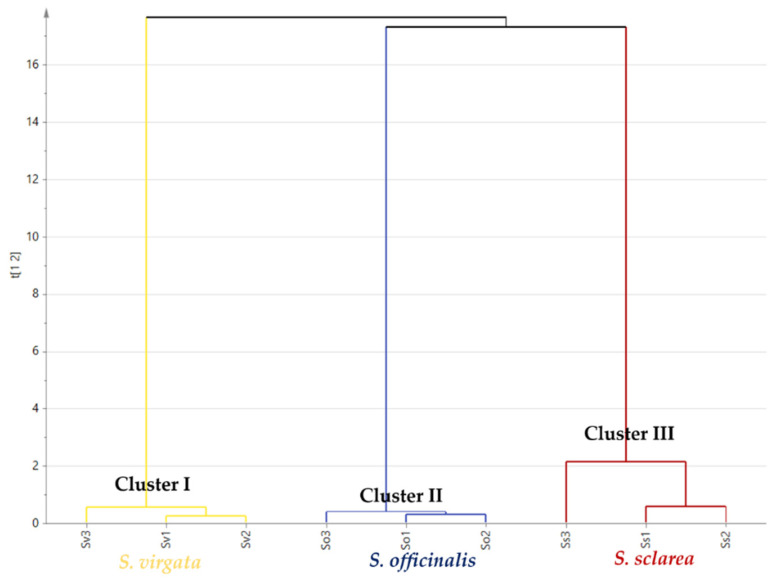
HCA dendrogram based on GC-MS chemical profile of the essential oils of different *Salvia* species as displayed in Table 1. *S. officinalis* (So), *S. virgata* (Sv) and *S. sclarea* (Ss).

Additionally, HCA was applied as an unsupervised pattern recognition method to support results obtained by PCA. Figure 2 shows the HCA dendrogram, which displays segregation of different *Salvia* species in three main clusters. Cluster I, II and III present *S. virgata* (Sv), *S. officinalis* (So) and *S. sclarea* (Ss), respectively. The HCA dendrogram reveals the closeness of *S. officinalis* (So) and *S. sclarea* (Ss). HCA results endorse that of PCA.

Partial least squares (PLS) was applied to find a correlation between the volatile compounds and their antioxidant and enzyme inhibitory activities. PLS-R1 and PLS-R2 models were constricted by the data matrix X containing the peak area of the GC/MS and the response y vectors containing the antioxidant and enzyme inhibitory activities data, respectively. The model performance was estimated by the parameters of root mean square error of calibration (RMSEC), root mean square error of validation (RMSEV) and correlation (R2). PLS-R1 model parameters, including slope, offset, RMSEC, RMSEV and R^2^, are shown in Table 4, indicating the strong prediction ability of the PLS regression model. PLS-R1 models showed excellent linearity and accuracy, with R^2^ > 0.99 and slope close to 1 (a value close to 1 means the predicted values are close to the reference), with low differences between RMSEC and root mean square error of validation (RMSEV) revealing the robustness of the model. It was observed that both DPPH and PBD data displayed the lowest RMSEV values (0.5325 and 0.6550), respectively, suggesting that they are more representative than other techniques to measure the antioxidant activity. The prediction performance for the developed models is shown in Table 5. The results show that the antioxidant activity is correctly predicted with ±5% accuracy.

Concerning PLS-R2, model parameters, including slope, offset, RMSEC, RMSEV and R2, are shown in Table 6, indicating the moderate prediction ability of the PLS regression model. PLS-R2 models showed good linearity and accuracy with R^2^ > 0.97, except for BChE inhibition, which exhibited much lower values. The prediction performance for the developed models is shown in Table 7.

## 3. Materials and Methods

### 3.1. Plant Material

The *S. officinalis* L. (LRR № 017; 14 May 2020) was cultivated in Uzbekistan and collected from the botanical field of the Institute of the Chemistry of Plant Substances (41°20′12.42″ N 69°20′06.07″ E, Tashkent, Uzbekistan). *S. virgata* Jacq. (LRR № 153; 25 June 2020) and *S. sclarea* L. (LRR № 095; 18 June 2020) were collected from Qizilsoy (41°12′11.6″ N 69°45′45.4″ E Tashkent region). The plants were identified by Olim Khojimatov and the voucher samples have been deposited at the National Herbarium of the Institute of Botany, Academy of Sciences of Uzbekistan.

### 3.2. Extraction of Essential Oils of Salvia Species

Aerial parts of *Salvia* samples were air-dried in the shade. Essential oils were hydro-distilled (400 g dry powder in 1 L distilled water) using a Clevenger-type apparatus for 3 h. The yields were 0.8% *w/w* for *S. officinalis,* 0.2% *w/w* for *S. virgata* and 0.3% *w/w* for *S. sclarea*. The recovered oils were dried over anhydrous sodium sulphate and kept in sealed dark vials at 4 °C until analysis.

### 3.3. GC-MS Analysis of Essential Oils of Salvia Species

GC-MS of *Salvia* essential oils was carried out using an Agilent 7890 B gas chromatograph (Agilent Technologies, Rotterdam, The Netherlands). The column used was a VF-Wax CP 9205 fused silica (30 m × 0.25 mm, ID 0.25 µm). Helium was used as carrier gas at a flow rate of 0.9 mL/min. An Agilent 5977A mass selective detector was used, with a scan range of 45–950 atomic mass units with a detector temperature of 270 °C and split mode injection at a split ratio of 1:20. An autosampler was used for sample injection (0.5 µL) with an injector temperature of 250 °C. The interface temperature was 280 °C, the source temperature was 230 °C, and the ionization energy was 70 eV. The initial oven temperature was 50 °C for 5 min., which was then raised to 280 °C at a rate of 5 °C/min, then kept isothermal at 280 °C for 15 min. Standard alkanes (C7-C40) obtained from Sigma-Aldrich (Darmstadt, Germany) were used to calculate the Kovats index (KI). Chromatograms were generated using enhanced ChemStation software (Agilent Technologies, Waldbronn, Germany). Volatile compounds were identified by comparing their mass spectra and KI was calculated with the 9th edition of Wiley Registry of mass spectral data and NIST library.

### 3.4. Antioxidant Assays

In vitro assays were employed to evaluate the antioxidant activity of the three *Salvia* EOs using the 2,2-diphenyl-1-picryl-hydrazyl (DPPH), 2,2′-azino-bis(3-ethylbenzothiazoline-6-sulfonic acid) (ABTS) radical-cation-based assay, total antioxidant capacity using cupric-reducing antioxidant capacity assay (CUPRAC), ferric-reducing antioxidant power assay (FRAP), EDTA chelating activity and phosphomolybdenum (PBD) assay. These assays were performed according to previously described standard procedures, and values are expressed as Trolox or EDTA equivalent [49,50]. The experimental procedures are given in supplemental materials. To provide a comparison with standard compounds, IC_50_ values (the half inhibitory concentration) were also calculated for DPPH, ABTS and metal chelating assays. IC_50_ values for other assays (reducing power and phosphomolybdenum) reflect that the concentration at which absorbance occurs is 0.5.

### 3.5. Enzyme Inhibitory Assays

The enzyme inhibitory effect of the oils was assessed against five enzymes which play a crucial step in certain medical conditions. These included AChE, BChE, tyrosinase, α-glucosidase and α-amylase. Assays were carried out according to standard procedures, with values expressed as galantamine, kojic acid and acarbose equivalent for cholinesterase, tyrosinase and α-glucosidase/α-amylase inhibitory activities, respectively [50,51]. The experimental procedures are given in supplemental materials. IC_50_ values (the half inhibitory concentration) for each oil and standard inhibitors were also calculated for enzyme inhibitory assays.

### 3.6. Statistical Analysis

All analyses were conducted in triplicate. Values are expressed as means ± SD. Statistical significance was determined using one-way ANOVA followed by Tukey’s post hoc test (significance level at *p* < 0.05).

### 3.7. Chemometric Analysis

The data obtained from GC-MS were subjected to chemometric analysis. Principal component analysis (PCA) was applied as an initial step for data investigation to present an overview of all species divergences and to recognize markers responsible for this dissimilarity [52]. Hierarchal cluster analysis (HCA) was then applied to allow the clustering of different species. The clustering pattern was constructed by the single linkage method. PCA and HCA were accomplished using the SIMCA-P version 13.0 software package (Umetrics, Umeå, Sweden). A quantitative calibration model, partial least squares (PLS), was designed to find a correlation between the volatile compounds (GC/MS peak areas) (X) matrix and their antioxidant, enzyme inhibitory activities (Y) matrices. In this state, there was no division of data into model and test set, as only nine samples for each model were assessed (small dataset). PLS was performed using CAMO’s Unscrambler^®^ X 10.4 software (Computer-Aided Modeling, AS, Oslo, Norway).

## 4. Conclusions

*Salvia* species are aromatic plants that have been widely used in various cultures since ancient times. In the present work, the chemical profile of three *Salvia* species essential oils was investigated. The studied species were *S. officinalis, S. virgata* and *S. sclarea.* Their major identified compounds were *cis*-thujone, 2,4-hexadienal and 9-octadecenoic acid in *S. officinalis, S. virgata* and *S. sclarea* EOs, respectively. The PCA score plot revealed significant discrimination of the three species even though its biplot was unable to identify the compounds responsible for these differences. The three *Salvia* species EOs exhibited moderate antioxidant activities. Highest AChE inhibitory activity was recorded for *S. officinalis*, while *S. virgata* had the highest BChE effect. All studied oils showed good tyrosinase inhibitory activity. Moreover, the oils did not exhibit any glucosidase inhibition, and exhibited weak or no activity as amylase inhibitors. Thus, the studied *Salvia* essential oils are interesting candidates that could be used in drug discovery for the management of Alzheimer’s and hyperpigmentation conditions.

## Figures and Tables

**Table 1:** Chemical profile of the aerial parts of *S. officinalis, S. virgata* and *S. sclarea* essential oils (*n* = 3 ± SD).

No	KI^†^		Compound	Relative Abundance %		
	Cal.	Rep.		*S. officinalis*	*S. virgata*	*S. sclarea*
**1**	1067	1067	Camphene	1.9	2.6	-
**2**	1130	1133	β-Thujene	0.9	-	-
**3**	1134	1137	β-Pinene	0.4	-	-
**4**	1148	1149	δ-3-Carene	-	0.6	-
**5**	1180	1184	β-Myrcene	0.9	1.2	0.8
**6**	1193	1195	α-Terpinene	-	3.0	-
**7**	1201	1203	Limonene	0.5	6.2	0.4
**8**	1207	1208	1,8-Cineole	8.9	-	1.2
**9**	1215	1216	(*E*)-2-Hexenal	0.2	1.6	0.4
**10**	1230	1232	γ-Terpinene	0.6	5.2	0.3
**11**	1238	1240	β-*trans*-Ocimene	-	-	0.5
**12**	1267	1268	*p*-Cymene	0.5	4.5	0.2
**13**	1274	1276	α-Terpinolene	0.3	3.0	tr
**14**	1296	1298	1-Octen-3-one	tr	-	-
**15**	1304	1305	2,4-Nonadienal	-	3.3	-
**16**	1340	1342	6-Methyl-5-heptene-2-one	-	0.2	-
**17**	1357	1359	1-Hexanol	-	0.2	-
**18**	1370	1372	*allo*-Ocimene	-	-	0.2
**19**	1390	1390	Nonanal	-	-	0.2
**20**	1391	1391	(*Z*)-Hex-3-en-1-ol	0.4	-	-
**21**	1394	1395	2,4-Hexadienal	-	9.4	-
**22**	1413	1414	Butyl hexanoate	-	tr	0.7
**23**	1427	1427	*trans*-2-Octenal	-	tr	-
**24**	1431	1435	*cis*-Thujone	18.6	0.9	0.7
**25**	1445	1445	*cis*-Linalool oxide	-	-	1.3
**26**	1447	1448	*trans*-Thujone	3.3	0.2	tr
**27**	1452	1453	1-Octen-3-ol	0.0	-	-
**28**	1455	1456	1-Heptanol	0.2	-	0.3
**29**	1464	1466	*trans*-Linalool oxide	0.4	0.2	1.1
**30**	1470	1470	Fenchyl acetate	-	tr	-
**31**	1484	1485	α-Campholenal	-	1.1	1.0
**32**	1493	1493	α-Copaene	-	0.8	1.3
**33**	1498	1498	*n*-Decanal	tr	0.3	0.2
**34**	1506	1505	Camphor	12.2	-	0.4
**35**	1521	1520	Benzaldehyde	-	0.4	0.7
**36**	1532	1532	(*Z*)-2-Nonenal	-	0.2	-
**37**	1553	1554	Linalool	0.4	0.2	-
**38**	1562	1564	Linalyl acetate	0.0	0.2	4.7
**39**	1565	1566	*trans*-Pinocamphone	tr	tr	0.3
**40**	1568	1569	(*E,E*)-3,5-Octadien-2-one	-	0.2	-
**41**	1573	1574	*Iso* pulegone	0.9	tr	0.2
**42**	1574	1574	Pinocarvone	0.2	0.2	-
**43**	1579	1579	Bornyl acetate	0.0	0.2	0.2
**44**	1581	1582	6-Methyl-3,5-heptadien-2-one	-	tr	0.3
**45**	1588	1589	*trans-*β-Caryophyllene	3.2	-	0.9
**46**	1599	1598	Terpinen-4-ol	0.9	1.3	tr
**47**	1608	1608	Aromadendrene	-	0.3	tr
**48**	1617	1619	Butyl octanoate	tr	-	0.9
**49**	1623	1624	β-Cyclocitral	tr	-	tr
**50**	1629	1628	1-Terpineol	tr	tr	0.2
**51**	1643	1644	Pulegone	-	0.5	0.2
**52**	1649	1650	Alloaromadendrene	1.0	0.2	0.3
**53**	1655	1658	Sabinyl acetate	0.3	-	tr
**54**	1672	1670	4-Vinylanisole	-	0.2	0.2
**55**	1677	1679	β-Citral	0.2	0.5	tr
**56**	1680	1681	α-Humulene	6.1	1.2	0.3
**57**	1684	1684	δ-Terpineol	0.2	-	0.5
**58**	1702	1703	γ-Muurolene	0.6	1.7	-
**59**	1711	1712	α-Terpineol	1.6	1.0	2.5
**60**	1714	1715	Borneol	4.0	0.3	0.2
**61**	1720	1722	Dodecanal	-	tr	0.2
**62**	1723	1725	Butyl nonanoate	-	1.0	0.3
**63**	1728	1729	Piperitone	0.5	1.7	0.2
**64**	1733	1733	Neryl acetate	tr	-	0.7
**65**	1742	1746	Carvyl acetate	tr	0.3	tr
**66**	1750	1750	Epoxylinalool	tr	-	0.2
**67**	1752	1752	δ-Cadinene	tr	-	1.4
**68**	1761	1763	1-Decanol	-	3.2	-
**69**	1782	1783	Cubenene	0.7	0.3	0.3
**70**	1784	1785	α-Cadinene	-	0.9	tr
**71**	1792	1793	Myrtenol	0.6	-	tr
**72**	1796	1797	2,4-Decadienal	-	tr	-
**73**	1803	1805	2-Tridecanone	-	tr	0.8
**74**	1814	1815	β-Damascenone	0.4	-	-
**75**	1822	1824	β-Damascone	-	2.2	0.3
**76**	1844	1845	*trans*-Calamenene	-	0.9	0.3
**77**	1855	1856	*cis*-Carveol	0.6	tr	-
**78**	1857	1857	*trans*-Carveol	tr	0.4	0.3
**79**	1867	1868	(*Z*)-Geranyl acetone	-	0.2	2.0
**80**	1869	1870	*exo*-2-Hydroxycineole	tr	tr	0.2
**81**	1884	1885	Benzyl alcohol	tr	0.2	0.4
**82**	1887	1887	(*E*)-2-Dodecenal	-	0.5	-
**83**	1915	1916	α-Calacorene	-	0.8	-
**84**	1917	1918	Piperitenone	tr	0.8	1.5
**85**	1920	1921	Tetradecanal	0.2	-	-
**86**	1926	1927	Phenylethyl alcohol	0.1	0.7	0.6
**87**	1930	1931	*trans*-β-Ionone	tr	0.3	0.3
**88**	1937	1938	*cis*-Jasmone	tr	0.2	-
**89**	1945	1945	2,6-Dimethyl-3,7-octadiene-2,6-diol	tr	0.2	0.3
**90**	1949	1951	(2*E*)-Hexenoic acid	0.4	0.2	0.6
**91**	1953	1955	*cis*-Caryophyllene oxide	0.2	tr	2.3
**92**	1954	1954	2-Ethyl-hexanoic acid	-	tr	0.4
**93**	1966	1967	β-Ionone epoxide	tr	0.5	0.3
**94**	1992	1993	*trans*-β-Ionone-5,6-epoxide	0.6	0.2	2.1
**95**	2000	2000	Eicosane	-	0.6	0.8
**96**	2000	2203	2-Methoxy-4-vinylphenol	tr	0.5	0.7
**97**	2001	2003	Methyl eugenol	0.5	-	0.3
**98**	2012	2014	Methyl tetradecanoate	0.2	tr	-
**99**	2022	2024	Glubulol	tr	0.4	tr
**100**	2030	2032	Cinnamaldehyde	1.2	tr	0.7
**101**	2034	2035	Nerolidol	tr	0.4	0.2
**102**	2041	2042	Pentadecanal	0.3	0.3	0.4
**103**	2051	2052	Octanoic acid	0.7	0.2	0.4
**104**	2080	2081	Viridiflorol	4.3	0.5	0.4
**105**	2095	2099	β-Elemenone	tr	0.9	tr
**106**	2121	2121	Spatulenol	tr	1.0	2.5
**107**	2130	2131	Hexahydrofarnesyl acetone	tr	0.5	0.4
**108**	2133	2135	2-Hydroxy-4-methoxy-benzaldehyde	-	0.4	0.2
**109**	2178	2179	γ-Eudesmol	-	0.5	0.2
**110**	2185	2186	Eugenol	tr	-	1.1
**111**	2192	2192	Nonanoic acid	0.3	0.7	0.6
**112**	2197	2198	Thymol	0.6	0.3	0.4
**113**	2205	2206	Carvacrol	0.7	0.5	0.2
**114**	2210	2210	Methyl hexadecanoate	-	0.3	0.5
**115**	2213	2215	β-Eudesmol	-	1.2	1.3
**116**	2217	2219	Ledene oxide-(I)	-	tr	0.7
**117**	2220	2223	Sclareoloxide	1.3	-	1.5
**118**	2240	2241	Ethyl hexadecanoate	0.4	0.5	2.4
**119**	2262	2264	*n*-Decanoic acid	tr	0.3	0.7
**120**	2300	2300	*n*-Tricosane	0.5	0.6	0.4
**121**	2321	2324	Dihydroactinolide	-	0.6	0.4
**122**	2330	2331	(6*R*)-(β)-Caryophyllene oxide	-	0.4	0.6
**123**	2340	2343	Octadecanal	0.5	-	3.4
**124**	2378	2379	4-Vinylphenol	tr	0.4	1.5
**125**	2389	2390	Isoelemicin	0.5	-	0.7
**126**	2394	2396	Tetracosane	tr	-	0.2
**127**	2416	2419	Butyl hexadecanoate	-	-	0.2
**128**	2450	2451	Dodecanoic acid	-	0.3	0.2
**129**	2465	2469	Penatcosane	tr	tr	0.3
**130**	2541	2545	Vanillin	-	0.9	0.3
**131**	2547	2550	9,12,15-Octadecatrienoic acid, methyl ester	-	tr	tr
**132**	2595	2597	*n*-Hexacosane	-	-	0.3
**133**	2650	2650	Benzyl benzoate	tr	-	tr
**134**	2654	2655	*n*-Butyl octadecanoate	5.6	0.2	5.7
**135**	2700	2712	*n*-Heptacosane	tr	0.5	0.4
**136**	2819	2819	Pentadecanoic acid	-	-	-
**137**	2826	2828	*n*-Octacosane	-	tr	-
**138**	2896	2899	*n*-Hexadecanoic acid	-	1.7	0.4
**139**	3000	3000	*n*-Triacontane	-	tr	0.4
**140**	3102	3100	*n*-Hentriacontane	-	tr	tr
**141**	3103	3104	Octadecanoic acid	-	tr	-
**142**	3153	3157	9-Octadecenoic acid	-	0.2	6.9
**143**	3165	3168	9,12-Octadecadienoic acid	-	0.2	tr
**144**	3192	3193	9,12,15-Octadecatrienoic acid	-	0.4	tr
**Total % of identified compounds**				**91.1**	**84.7**	**78.1**

Compounds were identified based on the compounds’ mass spectral data and retention indices compared with those of the NIST Mass Spectral Library (December 2011), the Wiley Registry of Mass Spectral Data, 8th edition, and many authentic standards. The content (%) was calculated in triplicate using the normalization method based on the GC-MS data. The presented data are the average of three replicas, tr—trace concentration less than 0.1%. Standard deviation did not exceed 3% for all values. KI**^†^**: Kovats index calculated on VF-Wax CP 9205column.

**Table 2:** Antioxidant activity of *Salvia* essential oils *.

Samples	DPPH		ABTS		CUPRAC		FRAP		Chelating		PBD	
	(mg TE/g oil)	IC_50_	(mg TE/g oil)	IC_50_	(mg TE/g oil)	IC_50_	(mg TE/g oil)	IC_50_	(mg EDTAE/g oil)	IC_50_	(mmol TE/g oil)	IC_50_
*S. officinalis*	NA	NA	55.7 ± 2.99	2.56 ± 0.19	74.3 ± 1.78	1.44 ± 0.01	55.3 ± 0.89	0.80 ± 0.02	62.3 ± 1.42	0.60 ± 0.01	26.4 ± 0.16	0.10 ± 0.01
*S. virgata*	26.6 ± 1.60	1.98 ± 0.23	190.1 ± 2.04	0.75 ± 0.01	275.2 ± 8.50	0.39 ± 0.02	155.9 ± 1.33	0.28 ± 0.01	NA	NA	15.1 ± 0.03	0.18 ± 0.01
*S. sclarea*	NA	NA	42.6 ± 0.51	3.34 ± 0.06	83.6 ± 0.72	1.28 ± 0.01	69.1 ± 2.02	0.64 ± 0.03	65.8 ± 2.85	0.58 ± 0.02	14.6 ± 0.26	0.19 ± 0.01
Trolox	-	0.22 ± 0.01	-	0.65 ± 0.09	-	0.44 ± 0.02	-	0.17 ± 0.01	-	-	-	0.68 ± 0.01
EDTA	-		-	-	-	-	-	-	-	0.04 ± 0.01	-	-

* Values are reported as mean ± S.D. of three parallel measurements. IC_50_ values reported as mg/mL. TE: Trolox equivalent; EDTAE: EDTA equivalent; NA: not active.

**Table 3:** Enzyme inhibitory properties of the *Salvia* essential oils *.

Samples	AChE İnhibition		BChE İnhibition		Tyrosinase İnhibition		Amylase İnhibition		Glucosidase İnhibition	
	(mg GALAE/g oil)	IC_50_	(mg GALAE/g oil)	IC_50_	(mg KAE/g oil)	IC_50_	(mmol ACAE/g oil)	IC_50_	(mmol ACAE/g oil)	IC_50_
*S. officinalis*	4.3 ± 0.01	0.68 ± 0.01	12.0 ± 0.53	0.61 ± 0.03	128.4 ± 4.35	0.73 ± 0.01	0.7 ± 0.05	1.27 ± 0.07	NA	NA
*S. virgata*	NA	NA	12.1 ± 0.16	0.60 ± 0.01	94.0 ± 1.75	0.90 ± 0.01	0.1 ± 0.01	>5	NA	NA
*S. sclarea*	2.9 ± 0.01	1.01 ± 0.01	11.5 ± 0.10	0.63 ± 0.01	66.1 ± 0.61	1.27 ± 0.07	1.1 ± 0.03	0.96 ± 0.01	NA	NA
Galantamine	-	0.01 ± 0.001	-	0.02 ± 0.01	-	-	-	-	-	-
Kojic acid	-	-	-	-	-	0.75 ± 0.01	-	-	-	-
Acarbose	-	-	-	-	-	-	-	0.66 ± 0.01	-	0.58 ± 0.01

* Values are reported as mean ± S.D. of three parallel measurements. IC_50_ values reported as mg/mL. GALAE: galantamine equivalent; KAE: kojic acid equivalent; ACAE: acarbose equivalent; NA: not active.

**Table 4:** PLS-R1 model parameters used for prediction.

Antioxidant Activity	Data Type	PLS-R1			
		Slope	Offset	RMSE	R^2^
DPPH	Cal.	0.9992	0.0066	0.3428	0.9992
	Val.	0.9959	0.0432	0.5325	0.9985
ABTS	Cal.	0.9998	0.0121	0.7539	0.9998
	Val.	0.9969	0.3342	1.1553	0.9997
FRAP	Cal.	0.9988	0.1088	1.5135	0.9988
	Val.	0.9959	0.4324	2.2877	0.9978
CUPRAC	Cal.	0.9996	0.0525	1.7693	0.9996
	Val.	0.9968	0.5267	2.6944	0.9993
EDTA	Cal.	0.9990	0.0414	0.9411	0.9990
	Val.	0.9959	0.1521	1.4157	0.9982
PBD	Cal.	0.9937	0.1166	0.4353	0.9937
	Val.	0.9874	0.2277	0.6550	0.9887

RMSE: root mean squared error. R^2^: correlation. Cal: calibration. Val: validation.

**Table 5:** Results of calibration and predictive ability of the PLS-R1 model. (*S. officinalis* (So), *S. virgata* (Sv) and *S. sclarea* (Ss)).

	DPPH		ABTS		FRAP	
	Y Reference	Y Predicted	Y Reference	Y Predicted	Y Reference	Y Predicted
**So1**	0.00	0.00	55.70	56.22	55.30	55.08
**So2**	0.00	0.00	56.80	56.52	54.70	55.17
**So3**	0.00	0.00	57.10	56.87	55.90	55.67
**Sv1**	25.90	26.72	191.60	191.62	155.90	155.87
**Sv2**	26.60	26.13	190.10	188.53	156.70	153.84
**Sv3**	27.5	27.13	192.40	193.87	154.50	157.27
**Ss1**	0.00	0.00	42.60	42.47	71.10	69.58
**Ss2**	0.00	0.00	42.90	43.14	68.50	69.95
**Ss3**	0.00	0.00	41.80	41.72	69.10	69.23
	**CUPRAC**		**EDTA**		**PBD**	
	**Y Reference**	**Y Predicted**	**Y Reference**	**Y Predicted**	**Y Reference**	**Y Predicted**
**So1**	74.30	73.88	63.50	62.61	26.40	26.38
**So2**	75.20	74.17	62.30	62.49	25.80	26.42
**So3**	73.50	74.99	61.60	62.29	26.90	26.27
**Sv1**	274.88	275.83	0.00	0.00	14.80	15.08
**Sv2**	275.20	271.55	0.00	0.00	15.10	15.19
**Sv3**	276.30	278.85	0.00	0.00	15.40	15.03
**Ss1**	82.50	83.46	65.80	65.54	14.60	14.41
**Ss2**	83.60	84.30	66.50	65.25	14.90	14.42
**Ss3**	84.20	82.60	64.40	65.86	13.70	14.36

**Table 6:** PLS-R2 model parameters used for prediction.

Enzyme Inhibition	Data Type	PLS-R2			
		Slope	Offset	RMSE	R^2^
AChE Inhibition	Cal.	0.9983	0.0039	0.0717	0.9983
	Val.	0.9948	0.0095	0.1062	0.9970
BChE Inhibition	Cal.	0.5769	5.0525	0.3346	0.5769
	Val.	0.3626	7.6161	0.5017	0.2483
Tyrosinase Inhibition	Cal.	0.9990	0.0894	0.7721	0.9990
	Val.	0.9947	0.4708	1.1165	0.9984
Amylase Inhibition	Cal.	0.9885	0.0076	0.0484	0.9885
	Val.	0.9792	0.0134	0.0732	0.9792

RMSE: root mean squared error. R^2^: correlation. Cal: calibration. Val: validation.

**Table 7:** Results of calibration and predictive ability of the PLS-R2 model.

	AChE Inhibition		BChE Inhibition	
	Y Reference	Y Predicted	Y Reference	Y Predicted
**So1**	0.00	0.00	55.70	56.22
**So2**	0.00	0.00	56.80	56.52
**So3**	0.00	0.00	57.10	56.87
**Sv1**	25.90	26.72	191.60	191.62
**Sv2**	26.60	26.13	190.10	188.53
**Sv3**	27.5	27.13	192.40	193.87
**Ss1**	0.00	0.00	42.60	42.47
**Ss2**	0.00	0.00	42.90	43.14
**Ss3**	0.00	0.00	41.80	41.72
	**Tyrosinase Inhibition**		**Amylase Inhibition**	
	**Y Reference**	**Y Predicted**	**Y Reference**	**Y Predicted**
**So1**	74.30	73.88	63.50	62.61
**So2**	75.20	74.17	62.30	62.49
**So3**	73.50	74.99	61.60	62.29
**Sv1**	274.88	275.83	0.00	0.00
**Sv2**	275.20	271.55	0.00	0.00
**Sv3**	276.30	278.85	0.00	0.00
**Ss1**	82.50	83.46	65.80	65.54
**Ss2**	83.60	84.30	66.50	65.25
**Ss3**	84.20	82.60	64.40	65.86

## Data Availability

Data are available upon request from the first author.

## Supplementary Materials

The following supporting information can be downloaded at: https://www.mdpi.com/article/10.3390/molecules27175365/s1, Figure S1: GC-chromatograms of the essential oils obtained from (A): *Salvia officnalis*, (B): *S. sclarea*, and (C): *S. virgata* aerial parts using the VF-Wax CP 9205 column.

## Sample Availability

Samples of the compounds are available from the authors.
