# Supplementary material for: GC-MS Chemical Profiling, Biological Investigation of Three Salvia Species Growing in Uzbekistan
Source: Molecules. 2022 Aug 23;27(17):5365. doi: 10.3390/molecules27175365 (PMC9457740; doi:10.3390/molecules27175365)
Supplement: Supplementary file 1 [file molecules-27-05365-s001.zip › molecules-1817545-supplementary.pdf]

Supplementary data

GC-MS chemical profiling, biological investigation of three *Salvia* species growing in Uzbekistan

Haidy A. Gad [1], Rano Z. Mamadalieva [2], Noha Khalil [3], Gokhan Zengin [4], Basma Najar [5], Olim K. Khojimatov [6], Nawal M. Al Musayeib [7], Mohamed L. Ashour [1*], Nilufar Z. Mamadalieva [6,8*]

Figure S1: GC-chromatograms of the essential oils obtained from (**A**): *Salvia officnalis,* (**B**): *S. sclarea,* and (**C**): *S. virgata* aerial parts using the VF-Wax CP 9205 column.

(A)

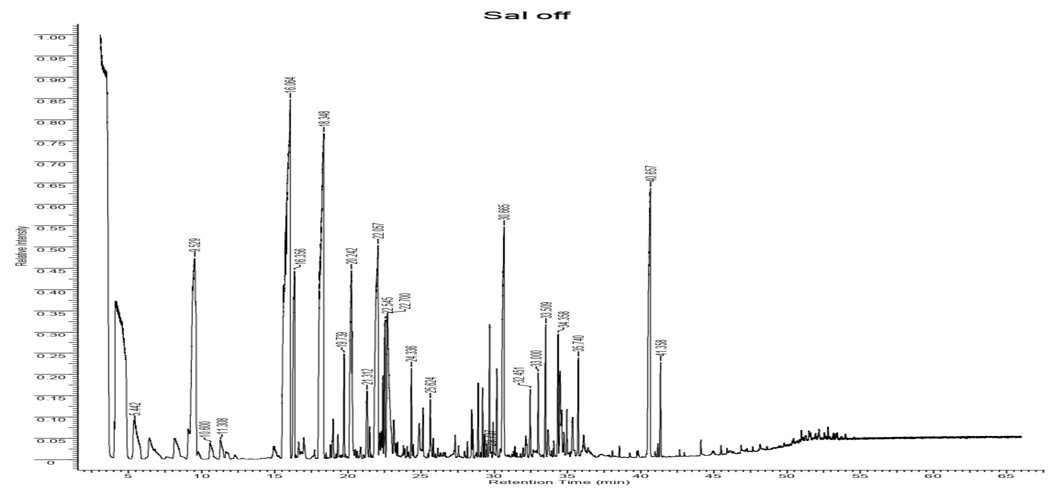
Sal off
Relative Intensity
Retention Time (min)
16.084
18.348
9.529
20.242
22.057
30.665
40.657
41.358
35.740
34.558
33.509
33.000

(B)

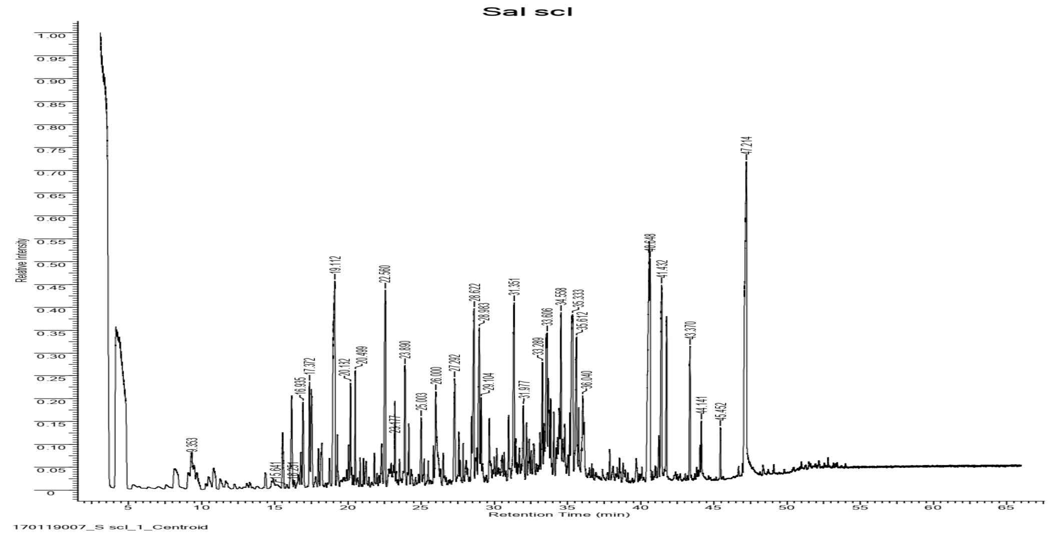
Sal scl
Relative Intensity
Retention Time (min)
170119007_S scl_1_Centroid
9.353
16.935
17.372
19.112
20.132
20.499
22.560
23.890
26.000
27.292
28.622
28.983
31.351
33.289
33.606
34.558
35.333
36.040
41.432
43.370
44.141
45.452
47.214

(C)

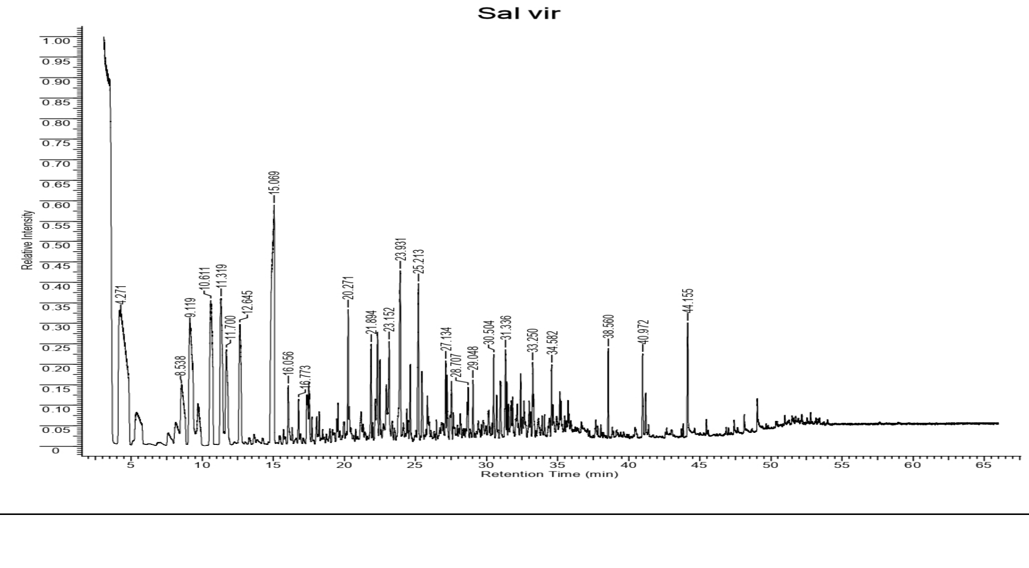
Sal vir
Relative Intensity
Retention Time (min)
4.271
8.538
9.119
10.611
11.319
11.700
12.645
15.069
16.056
16.773
20.271
21.894
23.152
23.931
25.213
27.134
28.707
29.048
30.504
31.336
33.250
34.582
38.560
40.972
44.155
